# The Role of Gastric Mucosal Immunity in Gastric Diseases

**DOI:** 10.1155/2020/7927054

**Published:** 2020-07-24

**Authors:** Siru Nie, Yuan Yuan

**Affiliations:** ^1^Tumor Etiology and Screening Department of Cancer Institute and General Surgery, The First Hospital of China Medical University, Shenyang 110001, China; ^2^Key Laboratory of Cancer Etiology and Prevention in Liaoning Education Department, The First Hospital of China Medical University, Shenyang 110001, China; ^3^Key Laboratory of GI Cancer Etiology and Prevention in Liaoning Province, The First Hospital of China Medical University, Shenyang 110001, China

## Abstract

Gastric mucosa plays its immune function through innate and adaptive immunity by recruiting immune cells and releasing corresponding cytokines, which have an inseparable relationship with gastric diseases. Whether infective gastric diseases caused by Helicobacter pylori, Epstein-Barr virus or other microbe, noninfective gastric diseases, or gastric cancer, gastric mucosal immunity plays an important role in the occurrence and development of the disease. Understanding the unique immune-related tissue structure of the gastric mucosa and its role in immune responses can help prevent gastric diseases or treat them through immunotherapy. In this review, we summarize the basic feature of gastric mucosal immunity and its relationship with gastric diseases to track the latest progress of gastric mucosal immunity, update relevant knowledge and provide theoretical reference for the prevention and treatment of gastric diseases based on the gastric mucosal immunity.

## 1. Introduction

The human body exerts immune responses through innate and adaptive immunity. After the invasion of pathogens, innate immunity acts as the first line of immune defense by recruiting innate immune cells, which belongs to natural nonspecific immunity. Adaptive immunity acts as a process of preventing infection by recruiting immune lymphocytes and producing immunoglobulins, which belongs to specific immunity. Gastrointestinal mucosal immune system is an important immune organ of the human body and exerts the same immune response as the human body [1]. However, some scholars reject the stomach as part of the gastrointestinal mucosal immune system, considering there is no mucosa-associated lymphoid tissue (MALT) in the gastric mucosa [2, 3]. With the deepening of research, it is now believed that the gastric mucosa can play its immune function in a layer-by-layer progressive mode through innate and adaptive immunity [4] and maintain the balance of microbe in an immune homeostasis mechanism [5]. On the one hand, when pathogens such as bacteria and viruses invade the gastric mucosa, both epithelial cells and innate immune cells begin to defend them through physical, chemical and biological processes. On the other hand, cytokines such as interleukin and chemokines secreted by immune cells help present antigens to lymphocytes such as T cells and B cells through antigen presentation, further triggering adaptive immunity. Understanding the unique immune-related tissue structure of the gastric mucosa and its role in immune responses can help prevent gastric diseases or treat them through immunotherapy. In this review, we will describe the basic feature of gastric mucosal immunity and its relationship with gastric diseases to track the latest progress of gastric mucosal immunity, update relevant knowledge and provide theoretical reference for the prevention and treatment of gastric diseases based on the gastric mucosal immunity.

## 2. Basic Features of Gastric Mucosal Immune System (Composition and Function)

The gastric mucosa is the inner layer of the gastric wall, which can be divided into three layers in histology: epithelial layer, lamina propria, and mucosal muscle layer. The gastric mucosa exerts different physiological functions through substances secreted by cells in different layers. Under normal circumstances, the lamina propria of the gastric mucosa does not have the same diffuse lymphoid tissue as intestinal mucosa and it does not have immune cells that immune directly. When the gastric mucosa is infected, immune cells are recruited to the gastric mucosa through a complex process, in which chemokines play an important role [6]. When antigens contact with the human body, immune cells in the blood will interact with activated vascular endothelial cells, slow down the movement of cells in the blood, and induce them to roll along the vessel wall. During this rolling process, the combination of immune cells and chemokines induces immune cells to adhere to the cell adhesion factor of endothelial cells via integrins and then migrate across the endothelial cells to the stomach [7]. After the recruitment of immune cells, many immune-related cells gather in the lamina propria of the gastric mucosa and play an important role in the subsequent inflammation and immune response together with natural epithelial barrier of the gastric mucosa.

### 2.1. Gastric Mucosal Innate Immunity-Associated Cells

Gastric mucosal innate immunity-associated cells consist of gastric mucosal epithelial cells, macrophages, dendritic cells (DCs), etc. Gastric mucosal epithelial cells locate in the epithelium of the gastric mucosa as the first line of defensing in the gastric mucosal immunity. Since gastric mucosal epithelial cells can express the major class II histocompatibility complex (MHC-II), we can consider it as an antigen-presenting cell (APC) participating in the initiation of innate immune response, which plays an important role in immune alert [8–10]. Other immune cells (such as macrophages, (DCs and natural killer cells) recruited in the lamina propria of the gastric mucosa also play an important role in the gastric mucosal immunity [11]. Macrophage microaggregates are widely distributed in the gastric mucosa [12]. After activation, cytokines produced by macrophage stimulate the occurrence of immune response, play a role in immune regulation and also promote the occurrence of adaptive immune response [13, 14]. In 2010, Bimczok et al. first identified the presence of dendritic cells in human gastric mucosa and confirmed the role of DCs as key initiators of immune response [15]. Interestingly, only activated mature DCs can play the initial immune response to pathogens. In the process of activation and maturation, DCs are affected by pathogen-associated molecular patterns (such as lipopolysaccharide, flagellin and bacterial DNA [16, 17]) and gastric stroma [18]. After activation, mature DCs act as APCs to induce adaptive immune responses via Toll-like receptor (TLR) signaling by activating effector T and B cells [3, 7, 19–21]. Nature killer cells (NK cells) also play an important role in innate immunity [22, 23]. In addition, when the gastric mucosa is invaded by pathogens, lymphocytes can gather in the lamina propria of the gastric mucosa through the formation of gastric lymph follicles; neutrophils and eosinophils can perform the immune functions of phagocytosis of pathogens and inhibition of bacterial colonization by upregulating the expression of chemokines and chemokine receptors [19].

### 2.2. Gastric Mucosa Adaptive Immunity-Associated Cells

Gastric mucosa adaptive immunity-associated cells consist of T cells, B cells, and other immune lymphocytes and are closely related to the formation of immunoglobulin. In the cellular immunity of the gastric mucosa, CD4^+^ T cells and Treg cells play an important role [2, 24]. Previous studies in mice have reported that Th1 and Th17 of CD4^+^ T cells play a protective role under the intermediation of neutrophils to maintain the dynamic balance of the immune system [6, 24]. Activated CD4^+^ T cells can also recruit neutrophils by secreting cytokines such as IL-17 and interferon-*γ* (IFN-*γ)* and upregulate the expression of *β*-protection and antimicrobial peptides [6]. The humoral immunity of the gastric mucosa mainly involves B cells and immunoglobulins. With the joint participation of macrophages, Th cells and chemokine (such as CCL28), B cells exert humoral immune functions [6]. As for immunoglobulins, as early as 40 years ago, S. Baur et al. [25] confirmed the existence of IgA and IgG containing cells in the glands of gastric lamina propria by immunofluorescence staining, which was further confirmed by A.J. Knox et al. [26]. They also confirmed the presence of IgG autoantibodies in inflammatory cells of the gastric mucosa. Nowadays, we believe that secretory IgA (sIgA) and IgM system play an important role in humoral immunity. After local immune cells produce immunoglobulins, dimer IgA and pentamer IgM act as secretory receptors to pass across the tight epithelium of the gastric mucosa through the way of selective transport [27]. The production of IgA is the first line of this immune defense. When the first line is breached, IgG was produced in the local gastric mucosa as the second line of defense and the number increased as the increase of inflammation in the gastric mucosa. Previous studies revealed that the ratio of IgA to IgG cells in normal gastric mucosa was 6 : 1. Although IgM is difficult to evaluate, a high proportion of IgM cells can also be seen in the gastric mucosa [27].

### 2.3. Basic Functions of Gastric Mucosal Immunity

In accordance with the immune response of the human body, the gastric mucosal immunity also exerts through innate immunity and adaptive immunity synergistically. The innate immunity of the gastric mucosa recognizes and phagocytizes pathogens through immune cells recruited in the lamina propria of the gastric mucosa and corresponding cytokines, playing a role of early immune alert as the first line of immune defense. In the gastric mucosal innate immunity, related cells (such as macrophages and dendritic cells) act as APCs to present antigens and then stimulate the adaptive immunity, as the second line of immune defense. As for the gastric mucosal adaptive immunity infected by pathogens, on the one hand, mediums produced by immune cells play a protective role to the gastric mucosa. On the other hand, pathogens play a damaging role to the gastric mucosa by escaping immune responses. When the damage of the gastric mucosa cannot be repaired, the dynamic balance of gastric mucosa barrier will be broken, which further leads to gastric diseases.

## 3. Gastric Mucosal Immunity and Gastric Diseases

### 3.1. Gastric Mucosal Immunity and Helicobacter pylori (*H. pylori*) Infection-Associated Gastric Diseases

#### 3.1.1. Gastric Mucosal Immunity and H. pylori-Infective Gastritis

*H. pylori*-infective gastritis is a very remarkable clinical outcome of *H. pylori* infection, which is closely related to the gastric mucosal immunity. After the infection of *H. pylori*, many innate immune cells play an important role. For example, the *H. pylori* CagA^+^ chain can interact with DCs to help them secrete IL-23, therefore promoting the polarization of Th22 and the expression of IL-22 receptor 1 in gastric epithelial cells. Consequently, the increased release of IL-22 stimulates gastric epithelial cells to secrete CXCL2, which further suppresses the Th1 protective immune response and leads to the occurrence of gastritis [28]. Some studies suggest that pathogens phagocytized by macrophages can be released by *H. pylori* and the damage effect of *H. pylori* is gradually greater than the protection effect, which eventually causes the damage of the gastric mucosa [7]. Not only innate immune-related cells but also other cells and molecules are involved in the development of *H. pylori*-infective gastritis. Recent evidence reveals that CCR6^+^ Treg cells in *H. pylori*-infected area of the gastric mucosa are positively correlated with the inflammation degree of *H. pylori*-related gastritis, which may be involved in the process of immunosuppression [29]. *H. pylori* infection can activate nucleotide-binding oligomerization domain 1(NOD1) and unable to regulate inflammatory body activation through mucin MUC1, which is also involved in the molecular mechanism of *H. pylori* infectious gastritis [30].

#### 3.1.2. Gastric Mucosal Immunity and Gastric Ulcer

Gastric ulcer is another common clinical outcome of *H. pylori* infection. Different from the immune response in gastritis, gastric ulcer is closely related to the distribution of gastric T cell subpopulations, which mainly performs in the ratio difference of CD4^+^/CD8^+^ T cell in the gastric mucosa [31]. When gastric ulcer occurs, CD3^+^ T cells transform into CD4^+^ T cells and CD19^+^ B cells [32]. The number of CD3^+^ T cells in *H. pylori*-positive gastric ulcer patients is significantly higher than that in gastritis, while the number of CD19^+^ B cells has no significant change [31]. According to the results of flow cytometry and immunohistochemistry, the number of CD19^+^ B cells, CD4^+^ Th cells, and CD8^+^ Tc cells in the gastric mucosa of gastric ulcer patients was higher than that of non-gastric ulcer patients. In addition, gastric ulcer can promote the expression of upstream IL-8, IL-10, and IL-10 in chronic infection to inhibit the occurrence of adaptive immune responses and weaken the clearance effect on antigen [32].

#### 3.1.3. Gastric Mucosal Immunity and Gastric MALT Lymphoma

Gastric MALT Lymphoma (GML) is an adaptive immune response to the immune inflammatory stimulation caused by *H. pylori*. It is a disease highly mediated by immune and inflammatory response. *H. pylori* infection initially leads to chronic infections, causing lymphoid hyperplasia. Then, under the chronic infection of *H. pylori*, *H. pylori* CagA is transferred from the human body to human B lymphocytes via type 4 secretion system encoded by cagPAI. After entering the cytoplasm, CagA binds to SHP-2 to form lymphoma by stimulating proliferation and inhibiting apoptosis of B lymphocytes through regulating intracellular pathway. CagA can also inhibit the proliferation of B lymphocytes by inhibiting the JAK-STAT signal pathway, helping bacteria escape from human body's specific immune response [33]. Moreover, data from several sources have identified the key role of directed mutation of immunoglobulin (Ig) heavy chain genes and the continuous activation of gene enhancer in the immune response of GML [34]. The involvement of Treg cells, BCR, and some cytokines (such as IL-22) was also confirmed in GML [35]. Recent evidence suggests that the chronic infection of *H. pylori* may cause GML, in which low-grade GML can possibly be cured by antibiotic therapy through eradicating *H. pylori* [36, 37].

#### 3.1.4. The Pathophysiological Mechanism of Gastric Mucosal Immune-Related H. pylori Infection

The gastric mucosa is protected and damaged by innate and adaptive immune responses after *H. pylori* infection, but gastric diseases will not occur in the early stage of infection. Therefore, the immune mechanism of *H. pylori* infection in the gastric mucosa is worth exploring. The study of the gastric mucosal immunity in mice has shown the recruitment of eosinophils and CD4^+^ T cells in the lamina propria of the gastric mucosa with increasing cell number as the increasing time of infection [6]. The large accumulation of immune cells and release of cytokines in the gastric mucosa offer a help to the activation of adaptive immune response [6, 38, 39]. In the gastric mucosal innate immunity associated with *H. pylori* infection, TIFA complex acting as the core regulatory factor and human *β*-defensin acting as the main component of the innate immune defense mechanism both stimulate the occurrence of adaptive immune response [40, 41]. In the gastric mucosal adaptive immunity associated with *H. pylori* infection, *H. pylori* initiates the immune response by recognizing pathogen-related molecular pattern through the pattern recognition receptor on gastric epithelial cells and innate immune cells [42] with the occurrence of Th1- and CD4^+^ T cell-related response (Th17 and Tregs response), the infiltration of corresponding immune cells and the participation of TNF-*α* signal pathway [32, 38, 43]. *H. pylori* can lead to the formation of gastric lymph follicles and the aggregation of lymphocytes in the lamina propria and induce the adaptive immune response by promoting the expression of T cells and B cells with the ability of adsorbing cell adhesion molecule-1 and attracting chemokines (CXCL10 and CCL28) [19].

At present, it is considered that *H. pylori* has both protective and damaging effects on mucosal immunity. Th1 and Th2 immune responses, along with cytokines and transcription regulators produced in the immune process, play an important role in the protective immune process of the gastric mucosa [32, 44, 45]. For example, the expression of chemokines CXCL1, CXCL2, and CXCL5 and their common receptor CXCR2 will increase to recruit neutrophils after *H. pylori* infection. The recruited neutrophils can kill *H. pylori* by directly phagocytosing bacteria or releasing active intermediates to resist *H. pylori* colonization and protect the gastric mucosa [6]; the expression of IFN-regulatory factor 8 and diversified immune-related gene characteristics of IL-11/STAT3 in the gastric mucosa are related to the inhibition of *H. pylori* colonization [45]. However, when the damage of the gastric mucosa cannot be completely repaired by the gastric mucosal immunity, *H. pylori* will smartly escape the immune response to cause further damage to the gastric mucosa, which is called immune escape. In this process, *H. pylori* escapes from immune response by activating inflammation and TLR cell signal pathways and changing surface molecules to avoid the recognition of innate immune receptors (such as TLRs and RIG-I of DCs) [38, 42]. In addition, the infiltration of immune cells (Th1, Treg cells, etc.) and the expression of corresponding cytokines (such as CCR6 ligand, chemokine CCL20, IFN-*γ*, and tumor necrosis factor-*α*) increase. Chemical gradients produced by cytokines can regulate the expression of cell adhesion receptor-ligand pairs and promote the recruitment of leukocytes to the site of injury, which is beneficial for *H. pylori* to escape from the host's immune defense, causing chronic inflammation and even gastric cancer [19, 42, 46–48].

### 3.2. Gastric Mucosal Immunity and Epstein-Barr Virus (EBV) Infection-Associated Gastric Diseases

EBV infection is closely related to gastritis and gastric cancer [49]. EBV can directly inhibit the proliferation of T cells and the toxicity of natural killer cells to maintain the activity of virus in host cells and cause continuously damage to the gastric mucosa [50]. The gastric mucosa infected by EBV can express high levels of ncRNA (EBV-encoded RNA and BART), and EBV miRNA can keep the virus at a very low expression level by targeting regulation of viral gene expression to avoid being attacked by human body's immune response [50, 51]. Increased expression of EBV-related genes also promotes the infiltration of immune cells and IFN-*γ* [52]. According to the infiltration of lymph immune cells in cellular immune responses, Epstein-Barr virus-associated gastric carcinoma (EBVaGC) can be divided into three histological subtypes: lymphoepithelial neoplasia (LELC), Crohn's disease-like lymphoid response (CLR), and conventional adenocarcinoma (CA) [52]. During the recruitment of immune lymphocytes to the gastric mucosa in EBVaGC, the expression of EBVaGC-related genes plays an important role. For example, IL-1*β* overexpression can recruit nonspecific lymphocytes to prevent direct contact between EBV-specific cytotoxic T cells and tumor cells [52]; IFN-*γ* overexpression can inhibit the proliferation and activation of CTL and NK cells [53]; PD-L1 expansion can inhibit the proliferation of T cells and the release of cytokines by interacting with PD-L1 receptors on T cells, and they can also induce CTL apoptosis and promote the differentiation from CD4^+^ T cells into Treg cells [53]. If the above-mentioned reactive immune cells are recruited into the tumor site or the surrounding area of EBVaGC, it may play a role in prolonging the survival time of EBVaGC patients.

### 3.3. Gastric Mucosal Immunity and Other Microbial Infection-Associated Gastric Diseases

In recent years, it has been suggested that microbiota is closely related to the occurrence and development of gastric diseases, and the immunity status is an important determinant of gastric microbiota [54]. Under normal circumstances, gastric microbiota is mainly composed of Prevotella, Neisseria, Streptococcus, Fusobacteria, and other microorganisms [55]. However, under the influence of host factors (anatomical characteristics, physiological characteristics, gender, age, etc. [56, 57]), external factors (diet, drug treatment, etc. [58]), and environmental factors (race, geographical location, etc. [59]), the composition of microorganisms in the stomach will change. Interestingly, the normal or abnormal stomach function composed by various microorganisms is regulated by both innate and adaptive immune responses. For example, recent studies on Fusobacterium are very popular. According to the 16S rRNA gene sequencing result of gastric cancer tissue, the distribution of Fusobacterium in gastric cancer tissues is significantly different from the adjacent tissue [60, 61]. Some studies have shown that Fusobacterium can protect tumors from the kill of NK cells and tumor-infiltrating T cells; therefore, we have reason to believe that the change of microbiota also has a certain impact on the gastric mucosal immunity [62].

### 3.4. Gastric Mucosal Immunity and Noninfective Gastric Diseases

The noninfective gastric disease is mainly autoimmune gastritis. Autoimmune gastritis is a chronic gastritis characterized by gastric mucosa atrophy, which is closely related to the lack of intrinsic factors [63]. The main targets of autoimmune reaction are parietal cells and intrinsic factors, in which the parietal cell antibody (PCAs) can be found in the gastric mucosa of 90% patients with atrophic gastritis [64]. Treg cells also play an important role in autoimmune gastritis and maintain certain tolerance to gastric autoantibodies. Treg cells can control the response of Tfh cells (follicular helper T cells) and the production of autoantibodies caused by gastric H^+^-K^+^-ATP enzymes and internal factors. They can also produce immunosuppressive cytokines (IL-10, TGF-*β*, IL-35, etc.) to exert immunosuppressive effects [65]. Studies have shown that the occurrence of autoimmune gastritis in Treg-deficient mice is closely related to the Th2 immune response. The immune response allows eosinophils to penetrate from the submucosal layer to the deeper layer, accompanied by the decrease in parietal cells and the production of autoimmune antibodies, leading to serious CD^+^ T cell dependent-autoimmune gastritis [66].

### 3.5. Gastric Mucosal Immunity and Gastric Cancer

Immunosuppression is an important factor in the development of gastric cancer. In recent years, immunotherapy for immune checkpoints represented by anti-PD-1/PD-L1 comes into the public eyes and plays an important role in tumor immunotherapy [67]. Immune checkpoint inhibitors enable immune cells such as T cells to recognize and kill tumor cells by lifting the restrictions of immune system and tumor cell defense system. Understanding the role and regulation of the gastric mucosal immunity in the development of gastric cancer may provide valuable reference for the diagnosis and treatment of gastric cancer. On the one hand, the activation of the gastric mucosal innate immunity and the increase of inflammatory cell infiltration lead to the increased risk of gastric cancer. On the other hand, the gastric mucosal adaptive immunity prevents the body from clearing pathogens, helping the pathogen escape the host's immune defense, leading to aggravated gastric mucosal damage and further developing gastric cancer.

In innate immune response, NK cells can play the role of innate immune response, and its mutation is closely related to the risk of gastric cancer. The activity of NK cells by killer cell immunoglobulin-like receptor (KIR), whose gene mutations can cause the mutation of NK cell activity to aggravate the inflammatory reaction of the gastric mucosa and the occurrence of gastric mucosa damage, eventually leads to gastric cancer [22, 23]. NOD1 is a sensor of intracellular innate immunity. Studies have shown that the expression level of NOD1 and TRAF3 in gastric cancer is lower than that in noncancer tissues. When the activation of the NOD1-TRAF3 signal pathway is impaired, it will appear intestinal metaplasia in the gastric mucosa [68]. Some other studies have reported that genetic mutations or gene polymorphisms are closely related to the gastric mucosal immunity in gastric cancer. For example, a Japanese study found that mutations of RIPK2 gene (receptor-interacting serine/threonine kinase 2) (rs16900627 minor allele genotype) increased the aggregation of inflammatory cells by changing the innate immune response of the gastric mucosa, leading to the atrophy of the gastric mucosa and increased risks of intestinal gastric cancer [69]. When pathogens pass APCs (such as DCs), aggregate through lymphocyte subsets and present antigens to corresponding immature T cells through TLRs, the adaptive immune response is activated [49]. In this way, under the specific conditions of foreign antigen presentation and cytokine aggregation, different types of T cells prevent the body from the elimination of pathogens through the corresponding inflammatory response and secretion of immunosuppressive cytokines, resulting in chronic infection and disease. Th17 can induce the production of specific matrix metalloproteinases by releasing IL-17 and IL-21, causing the damage of the gastric mucosa [46]. There are also studies showing that B cells are active in gastric cancer and can reflect the immune status of the gastric mucosa [70].

In addition to immune cells and cytokines in the above processes, genes that involved in the carcinogenesis of gastric cancer also play an important role in immune regulation. For example, when the gastric mucosa is infected with *H. pylori*, the expression of GATA-binding protein 3, a transcription factor regulating adaptive immune response, is increased, which inhibits the normal expression of Cx43 and causes the gastric mucosa to undergo a malignant transformation from inflammation to tumor [71]. Another transcription factor, Foxp3, promotes tumor growth by suppressing IL-10- and/or TGF-*β*-mediated tumor cell killing after increased infiltration of Treg cells [72]. SLFN5, as an IFN-*α* regulatory gene in immune cells, is also associated with the occurrence of gastric cancer [73]. When the gastric mucosa is infected with EBV, as mentioned above, PD-L1 and other immune checkpoints are closely related to the occurrence and prognosis of EBVaGC.

## 4. Conclusions and Prospect

In summary, the various immune cells, cytokines, and signal pathways effecting in the process of the gastric mucosal immunity have an inseparable relationship with gastric diseases. Whether *H. pylori*, EBV and other pathogen infective-associated gastric diseases, or noninfective-associated gastric diseases, the gastric mucosal immunity plays an important role in the occurrence and development of the disease. However, the gastric mucosal immunity induced by pathogen is a dynamic balance between protective immunity and damage immunity, by which this specific balance function mechanism needs further study. Among many pathogens, the study of microbial flora is in a hot spot and its mechanism of gastric mucosal immune regulation for gastric diseases still needs to be explored. Meanwhile, in view of the rapid development of immunotherapy, whether gastric diseases can be treated through more perfect and accurate immune targets according to the research progress of the gastric mucosal immunity and the corresponding mechanism is still worth further exploration. At present, some gastric cancer immune checkpoint inhibitors (such as PD1/PD-L1 inhibitors) have entered Phase 2 and Phase 3 clinical trials [74, 75] and immunotherapy combined with chemotherapy or radiotherapy has the opportunity to be used as first-line treatment for gastric cancer [76, 77]. In the future, based on the theory of the gastric mucosal immunity, searching for specific tumor microenvironment-related indicators as biomarkers for assessing treatment efficacy will play an important role in precision immunotherapy of gastric diseases.

## References

[1] Ahluwalia B., Magnusson M. K., Ohman L. (2017). Mucosal immune system of the gastrointestinal tract: maintaining balance between the good and the bad. *Scandinavian Journal of Gastroenterology*.

[2] Michetti M., Kelly C. P., Kraehenbuhl J.–. P., Bouzourene H., Michetti P. (2000). Gastric mucosal *α*4*β*7-integrin—positive CD4 T lymphocytes and immune protection against Helicobacter infection in mice. *Gastroenterology*.

[3] Shiu J., Blanchard T. G. (2013). Dendritic cell function in the host response to Helicobacter pylori infection of the gastric mucosa. *Pathogens and Disease*.

[4] Zhang B., Ren J.-L. (2005). Research progress of gastric mucosal immune mechanism. *World Chin J Digestol*.

[5] Bimczok D., Kao J. Y., Zhang M. (2015). Human gastric epithelial cells contribute to gastric immune regulation by providing retinoic acid to dendritic cells. *Mucosal Immunology*.

[6] Flach C. F., Mozer M., Sundquist M., Holmgren J., Raghavan S. (2012). Mucosal vaccination increases local chemokine production attracting immune cells to the stomach mucosa of Helicobacter pylori infected mice. *Vaccine*.

[7] Ieni A., Barresi V., Rigoli L., Fedele F., Tuccari G., Caruso R. (2016). Morphological and cellular features of innate immune reaction in Helicobacter pylori gastritis: a brief review. *International Journal of Molecular Sciences*.

[8] Barrera C., Espejo R., Reyes V. E. (2002). Differential glycosylation of MHC class II molecules on gastric epithelial cells: implications in local immune responses. *Human Immunology*.

[9] Krauss-Etschmann S., Gruber R., Plikat K. (2005). Increase of antigen-presenting cells in the gastric mucosa of Helicobacter pylori-infected children. *Helicobacter*.

[10] Gall A., Gaudet R. G., Gray-Owen S. D., Salama N. R. (2017). TIFA signaling in gastric epithelial cells initiates thecagType 4 secretion system-dependent innate immune response toHelicobacter pyloriInfection. *mBio*.

[11] Moyat M., Velin D. (2014). Immune responses to Helicobacter pylori infection. *World Journal of Gastroenterology*.

[12] de Magalhães-Costa M. H., dos Reis B. R., Chagas V. L. A., Nunes T., de Souza H. S. P., Zaltman C. (2014). Focal enhanced gastritis and macrophage microaggregates in the gastric mucosa: potential role in the differential diagnosis between Crohn's disease and ulcerative colitis. *Arquivos de Gastroenterologia*.

[13] Kaparakis M., Walduck A. K., Price J. D. (2008). Macrophages are mediators of gastritis in acute Helicobacter pylori infection in C57BL/6 mice. *Infection and Immunity*.

[14] Gordon S. (2003). Alternative activation of macrophages. *Nature Reviews. Immunology*.

[15] Bimczok D., Clements R. H., Waites K. B. (2010). Human primary gastric dendritic cells induce a Th1 response to H. pylori. *Mucosal Immunology*.

[16] Tsujimura H., Tamura T., Kong H. J. (2004). Toll-like receptor 9 signaling activates NF-kappaB through IFN regulatory factor-8/IFN consensus sequence binding protein in dendritic cells. *Journal of Immunology*.

[17] Re F., Strominger J. L. (2001). Toll-like receptor 2 (TLR2) and TLR4 differentially activate human dendritic cells. *The Journal of Biological Chemistry*.

[18] Bimczok D., Grams J. M., Stahl R. D., Waites K. B., Smythies L. E., Smith P. D. (2011). Stromal regulation of human gastric dendritic cells restricts the Th1 response to Helicobacter pylori. *Gastroenterology*.

[19] Hansson M., Sundquist M., Hering S., Lundin B. S., Hermansson M., Quiding-Järbrink M. (2013). DC-LAMP+ dendritic cells are recruited to gastric lymphoid follicles in Helicobacter pylori-infected individuals. *Infection and Immunity*.

[20] Steinman R. M. (2012). Decisions about dendritic cells: past, present, and future. *Annual Review of Immunology*.

[21] Kranzer K., Söllner L., Aigner M. (2005). Impact of Helicobacter pylori virulence factors and compounds on activation and maturation of human dendritic cells. *Infection and Immunity*.

[22] Hafsi N., Voland P., Schwendy S. (2004). Human dendritic cells respond to Helicobacter pylori, promoting NK cell and Th1-effector responses in vitro. *Journal of Immunology*.

[23] Hernandez E. G., Partida-Rodriguez O., Camorlinga-Ponce M. (2018). Genotype B of _Killer Cell Immunoglobulin-Like Receptor_ is Related with Gastric Cancer Lesions. *Scientific Reports*.

[24] Carbo A., Bassaganya-Riera J., Pedragosa M. (2013). Predictive computational modeling of the mucosal immune responses during Helicobacter pylori infection. *PLoS One*.

[25] Baur S., Koo N., Taylor K. B. (1970). The immunoglobulin class of autoantibody-containing cells in the gastric mucosa. *Immunology*.

[26] Knox A. J., von Westarp C., Row V. V., Volpe R. (1976). Thyroid antigen stimulates lymphocytes from patients with Graves' disease to produce thyroid-stimulating immunoglobulin (TSI). *The Journal of Clinical Endocrinology and Metabolism*.

[27] Valnes K., Brandtzaeg P., Elgjo K., Stave R. (1986). Quantitative distribution of immunoglobulin-producing cells in gastric mucosa: relation to chronic gastritis and glandular atrophy. *Gut*.

[28] Zhuang Y., Cheng P., Liu X. F. (2015). A pro-inflammatory role for Th22 cells in Helicobacter pylori-associated gastritis. *Gut*.

[29] Wu Y. Y., Hsieh C. T., Tsay G. J. (2019). Recruitment of CCR6(+ ) Foxp3(+) regulatory gastric infiltrating lymphocytes in Helicobacter pylori gastritis. *Helicobacter*.

[30] Ng G. Z., Menheniott T. R., Every A. L. (2016). The MUC1 mucin protects against Helicobacter pylori pathogenesis in mice by regulation of the NLRP3 inflammasome. *Gut*.

[31] Goll R., Husebekk A., Isaksen V., Kauric G., Hansen T., Florholmen J. (2005). Increased frequency of antral CD4 T and CD19 B cells in patients with Helicobacter pylori-related peptic ulcer disease. *Scandinavian Journal of Immunology*.

[32] Goll R., Gruber F., Olsen T. (2007). Helicobacter pylori stimulates a mixed adaptive immune response with a strong T-regulatory component in human gastric mucosa. *Helicobacter*.

[33] Floch P., Megraud F., Lehours P. (2017). Helicobacter pylori strains and gastric MALT lymphoma. *Toxins*.

[34] Pereira M. I., Medeiros J. A. (2014). Role of Helicobacter pylori in gastric mucosa-associated lymphoid tissue lymphomas. *World Journal of Gastroenterology*.

[35] Laur A. M., Floch P., Chambonnier L. (2016). Regulatory T cells may participate in Helicobacter pylori persistence in gastric MALT lymphoma: lessons from an animal model. *Oncotarget*.

[36] Shirwaikar Thomas A., Schwartz M., Quigley E. (2019). Gastrointestinal lymphoma: the new mimic. *BMJ Open Gastroenterol*.

[37] Park J. B., Koo J. S. (2014). Helicobacter pylori infection in gastric mucosa-associated lymphoid tissue lymphoma. *World Journal of Gastroenterology*.

[38] Kronsteiner B., Bassaganya-Riera J., Philipson C. (2016). Systems-wide analyses of mucosal immune responses to Helicobacter pylori at the interface between pathogenicity and symbiosis. *Gut Microbes*.

[39] Raghavan S., Ostberg A. K., Flach C. F. (2010). Sublingual immunization protects against Helicobacter pylori infection and induces T and B cell responses in the stomach. *Infection and Immunity*.

[40] Zimmermann S., Pfannkuch L., Al-Zeer M. A. (2017). ALPK1- and TIFA-dependent innate immune response triggered by the Helicobacter pylori type IV secretion system. *Cell Reports*.

[41] Bauer B., Wex T., Kuester D., Meyer T., Malfertheiner P. (2013). Differential expression of human beta defensin 2 and 3 in gastric mucosa of Helicobacter pylori-infected individuals. *Helicobacter*.

[42] Karkhah A., Ebrahimpour S., Rostamtabar M. (2019). Helicobacter pylori evasion strategies of the host innate and adaptive immune responses to survive and develop gastrointestinal diseases. *Microbiological Research*.

[43] Thalmaier U., Lehn N., Pfeffer K., Stolte M., Vieth M., Schneider-Brachert W. (2002). Role of tumor necrosis factor alpha in Helicobacter pylori gastritis in tumor necrosis factor receptor 1-deficient mice. *Infection and Immunity*.

[44] Sakai K., Kita M., Sawai N. (2008). Levels of interleukin-18 are markedly increased in Helicobacter pylori-infected gastric mucosa among patients with specific IL18 genotypes. *The Journal of Infectious Diseases*.

[45] Menheniott T. R., Judd L. M., Giraud A. S. (2015). STAT3: a critical component in the response to Helicobacter pylori infection. *Cellular Microbiology*.

[46] Maleki Kakelar H., Barzegari A., Dehghani J. (2019). Pathogenicity of Helicobacter pylori in cancer development and impacts of vaccination. *Gastric Cancer*.

[47] Tsai H. F., Hsu P. N. (2010). Interplay between Helicobacter pylori and immune cells in immune pathogenesis of gastric inflammation and mucosal pathology. *Cellular & Molecular Immunology*.

[48] Razavi A., Bagheri N., Azadegan-Dehkordi F. (2015). Comparative immune response in children and adults with H. pylori infection. *Journal of Immunology Research*.

[49] Martínez-López J., Torres J., Camorlinga-Ponce M., Mantilla A., Leal Y., Fuentes-Pananá E. (2014). Evidence of Epstein-Barr virus association with gastric cancer and non-atrophic gastritis. *Viruses*.

[50] Polakovicova I., Jerez S., Wichmann I. A., Sandoval-Bórquez A., Carrasco-Véliz N., Corvalán A. H. (2018). Role of microRNAs and exosomes in Helicobacter pylori and Epstein-Barr virus associated gastric cancers. *Frontiers in Microbiology*.

[51] Albanese M., Tagawa T., Buschle A., Hammerschmidt W. (2017). MicroRNAs of Epstein-Barr virus control innate and adaptive antiviral immunity. *Journal of Virology*.

[52] Cho J., Kang M. S., Kim K. M. (2016). Epstein-Barr virus-associated gastric carcinoma and specific features of the accompanying immune response. *Journal of Gastric Cancer*.

[53] Strong M. J., Xu G., Coco J. (2013). Differences in gastric carcinoma microenvironment stratify according to EBV infection intensity: implications for possible immune adjuvant therapy. *PLoS Pathogens*.

[54] von Rosenvinge E. C., Song Y., White J. R., Maddox C., Blanchard T., Fricke W. F. (2013). Immune status, antibiotic medication and pH are associated with changes in the stomach fluid microbiota. *The ISME Journal*.

[55] Espinoza J. L., Matsumoto A., Tanaka H., Matsumura I. (2018). Gastric microbiota: an emerging player in Helicobacter pylori-induced gastric malignancies. *Cancer Letters*.

[56] Human Microbiome Project, C (2012). Structure, function and diversity of the healthy human microbiome. *Nature*.

[57] Husebye E., Skar V., Hoverstad T., Melby K. (1992). Fasting hypochlorhydria with gram positive gastric flora is highly prevalent in healthy old people. *Gut*.

[58] WILLIAMS C., McCOLL K. E. L. (2006). Review article: proton pump inhibitors and bacterial overgrowth. *Alimentary Pharmacology & Therapeutics*.

[59] Yu G., Hu N., Wang L. (2017). Gastric microbiota features associated with cancer risk factors and clinical outcomes: a pilot study in gastric cardia cancer patients from Shanxi, China. *International Journal of Cancer*.

[60] Chen X. H., Wang A., Chu A. N., Gong Y. H., Yuan Y. (2019). Mucosa-associated microbiota in gastric cancer tissues compared with non-cancer tissues. *Frontiers in Microbiology*.

[61] Hsieh Y. Y., Tung S. Y., Pan H. Y. (2018). Increased abundance of Clostridium and Fusobacterium in gastric microbiota of patients with gastric cancer in Taiwan. *Scientific Reports*.

[62] Abed J., Maalouf N., Parhi L., Chaushu S., Mandelboim O., Bachrach G. (2017). Tumor targeting by Fusobacterium nucleatum: a pilot study and future perspectives. *Frontiers in Cellular and Infection Microbiology*.

[63] Hall S. N., Appelman H. D. (2019). Autoimmune gastritis. *Archives of Pathology & Laboratory Medicine*.

[64] Negrini R., Savio A., Poiesi C. (1996). Antigenic mimicry between Helicobacter pylori and gastric mucosa in the pathogenesis of body atrophic gastritis. *Gastroenterology*.

[65] Bagheri N., Salimzadeh L., Shirzad H. (2018). The role of T helper 1-cell response in Helicobacter pylori-infection. *Microbial Pathogenesis*.

[66] Harakal J., Rival C., Qiao H., Tung K. S. (2016). Regulatory T cells control Th2-dominant murine autoimmune gastritis. *Journal of Immunology*.

[67] Park R., Williamson S., Kasi A., Saeed A. (2018). Immune therapeutics in the treatment of advanced gastric and esophageal cancer. *Anticancer Research*.

[68] Minaga K., Watanabe T., Kamata K., Asano N., Kudo M. (2018). Nucleotide-binding oligomerization domain 1 andHelicobacter pyloriinfection: a review. *World Journal of Gastroenterology*.

[69] Ota M., Tahara T., Otsuka T. (2018). Association between receptor interacting serine/threonine kinase 2 polymorphisms and gastric cancer susceptibility. *Oncology Letters*.

[70] Kunori T., Shinya F., Satomi T. (1996). Spontaneous antibody-secreting cells in the stomach of gastric cancer patients. *Journal of Gastroenterology*.

[71] Liu X., Cao K., Xu C. (2015). GATA-3 augmentation down-regulates Connexin43 in Helicobacter pylori associated gastric carcinogenesis. *Cancer Biology & Therapy*.

[72] Kindlund B., Sjöling Å., Yakkala C. (2017). CD4(+) regulatory T cells in gastric cancer mucosa are proliferating and express high levels of IL-10 but little TGF-*β*. *Gastric Cancer*.

[73] Nápoles O. C., Tsao A. C., Sanz-Anquela J. M. (2017). SCHLAFEN 5 expression correlates with intestinal metaplasia that progresses to gastric cancer. *Journal of Gastroenterology*.

[74] Kang Y. K., Boku N., Satoh T. (2017). Nivolumab in patients with advanced gastric or gastro-oesophageal junction cancer refractory to, or intolerant of, at least two previous chemotherapy regimens (ONO-4538-12, ATTRACTION-2): a randomised, double-blind, placebo-controlled, phase 3 trial. *Lancet*.

[75] Muro K., Chung H. C., Shankaran V. (2016). Pembrolizumab for patients with PD-L1-positive advanced gastric cancer (KEYNOTE-012): a multicentre, open-label, phase 1b trial. *The Lancet Oncology*.

[76] Kubota Y., Kawazoe A., Sasaki A. (2020). The impact of molecular subtype on efficacy of chemotherapy and checkpoint inhibition in advanced gastric cancer. *Clinical Cancer Research*.

[77] Taieb J., Moehler M., Boku N. (2018). Evolution of checkpoint inhibitors for the treatment of metastatic gastric cancers: current status and future perspectives. *Cancer Treatment Reviews*.

